# Edelman Revisited: Concepts, Achievements, and Challenges

**DOI:** 10.3389/fmed.2021.808765

**Published:** 2022-01-10

**Authors:** Mark Rohrscheib, Ramin Sam, Dominic S. Raj, Christos P. Argyropoulos, Mark L. Unruh, Susie Q. Lew, Todd S. Ing, Nathan W. Levin, Antonios H. Tzamaloukas

**Affiliations:** ^1^Department of Medicine, University of New Mexico School of Medicine, Albuquerque, NM, United States; ^2^Department of Medicine, Zuckerberg San Francisco General Hospital and Trauma Center, University of California San Francisco School of Medicine, San Francisco, CA, United States; ^3^Department of Medicine, George Washington University, Washington, DC, United States; ^4^Department of Medicine, Stritch School of Medicine, Loyola University Chicago, Maywood, IL, United States; ^5^Mount Sinai Icahn School of Medicine, New York, NY, United States; ^6^Research Service, Department of Medicine, Raymond G. Murphy Veterans Affairs Medical Center and University of New Mexico School of Medicine, Albuquerque, NM, United States

**Keywords:** dysnatremia, hyponatremia, hypernatremia, osmotic sodium inactivation, hydrophilic compounds

## Abstract

The key message from the 1958 Edelman study states that combinations of external gains or losses of sodium, potassium and water leading to an increase of the fraction (total body sodium plus total body potassium) over total body water will raise the serum sodium concentration ([Na]_S_), while external gains or losses leading to a decrease in this fraction will lower [Na]_S_. A variety of studies have supported this concept and current quantitative methods for correcting dysnatremias, including formulas calculating the volume of saline needed for a change in [Na]_S_ are based on it. Not accounting for external losses of sodium, potassium and water during treatment and faulty values for body water inserted in the formulas predicting the change in [Na]_S_ affect the accuracy of these formulas. Newly described factors potentially affecting the change in [Na]_S_ during treatment of dysnatremias include the following: (a) exchanges during development or correction of dysnatremias between osmotically inactive sodium stored in tissues and osmotically active sodium in solution in body fluids; (b) chemical binding of part of body water to macromolecules which would decrease the amount of body water available for osmotic exchanges; and (c) genetic influences on the determination of sodium concentration in body fluids. The effects of these newer developments on the methods of treatment of dysnatremias are not well-established and will need extensive studying. Currently, monitoring of serum sodium concentration remains a critical step during treatment of dysnatremias.

## Introduction

Dysnatremias contribute to morbidity, mortality, and high medical costs (1, 2). Hyponatremia represents the most frequently encountered electrolyte abnormality in both the general population (3, 4) and specific segments of the population [e.g., older adults, hospitalized individuals and patients with pre-dialysis chronic kidney disease (5)]. Currently, the evaluation of pathophysiology and the methods of management of dysnatremias are directly linked to Edelman's seminal work (6).

In recent years, new discoveries related to body sodium and water homeostasis have created additional insights to the concepts developed by Edelman. This report presents the key points of the methodology of the Edelman study, its main findings, its application to the analysis of pathophysiology and to the methods of management of dysnatremias, and the opportunities created by newer findings.

## Review

### The Edelman Study

#### Design and Main Findings of the Study

Edelman and coinvestigators explored the hypothesis that sodium concentration in serum water ([Na]_SW_) is a function of total body exchangeable sodium (TBNa), total body exchangeable potassium (TBK), and total body water (TBW). This hypothesis implies equal solute concentrations across cell membranes, with sodium salts representing the extracellular solutes and potassium salts representing the intracellular solutes, and free movement of water across cell membranes, so that solute concentration is equal in the intracellular and extracellular compartments.

The Edelman study analyzed the determinants of [Na]_SW_ in 98 patients with diseases of the heart, liver, kidneys, lungs, gastrointestinal system, central nervous system, and miscellaneous other disease states affecting body water and sodium homeostasis (6). Several patients in Edelman's study had edema. TBW was measured by dilution of deuterium oxide, with an equilibration time of 6 h. Relatively rapid exchangeable TBNa and TBK were measured by dilution of radioisotopes (Na^24^–equilibration time 24 h—and K^24^–equilibration time 40 h—, respectively). Serum sodium concentration ([Na]_S_) was measured by flame photometry. Serum water content was measured by drying in prolonged heat one mL aliquots of serum, and [Na]_SW_ was calculated from [Na]_S_ and serum water content (SWC). Serum osmolality was measured by freezing point depression. The concentrations in serum of glucose (range 3.7–27.9 mmol/L or 66–502 mg/dL) and non-protein nitrogen (9–238 mg/dL) were used to correct osmolality when the relation between osmolality and [Na]_SW_ was analyzed.

[Na]_SW_, which varied between 108.9 and 192.5 mmol/L, was compared to serum osmolality, TBNa, TBK, and TBW by linear regression. The analyses of the study provided support for the following concepts: (a) Serum sodium salts account for the majority of serum osmolality. (b) [Na]_SW_ provides no information about TBNa, TBK, or TBW individually. (c) However, the relationship between TBNa, TBK, and TBW determines [Na]_SW_. This third concept represents the confirmation of the hypothesis of the Edelman study. The relationship between TBNa, TBK, and TBW was expressed by regression formula 1 (6):


(1) $$\begin{array}{l} {\left[Na\right]}_{SW}\;=1.11\times \frac{TBNa\;+\;TBK}{TBW}-\;25.6\;\text{r}=0.83 \end{array}$$

Subsequently, Boling and Lipkind verified the fundamental Edelman concept in a study performed by essentially the same methods (7). The range of [Na]_S_ in their study was 136.7–155.9 mmol/L.

**Figure 1:**
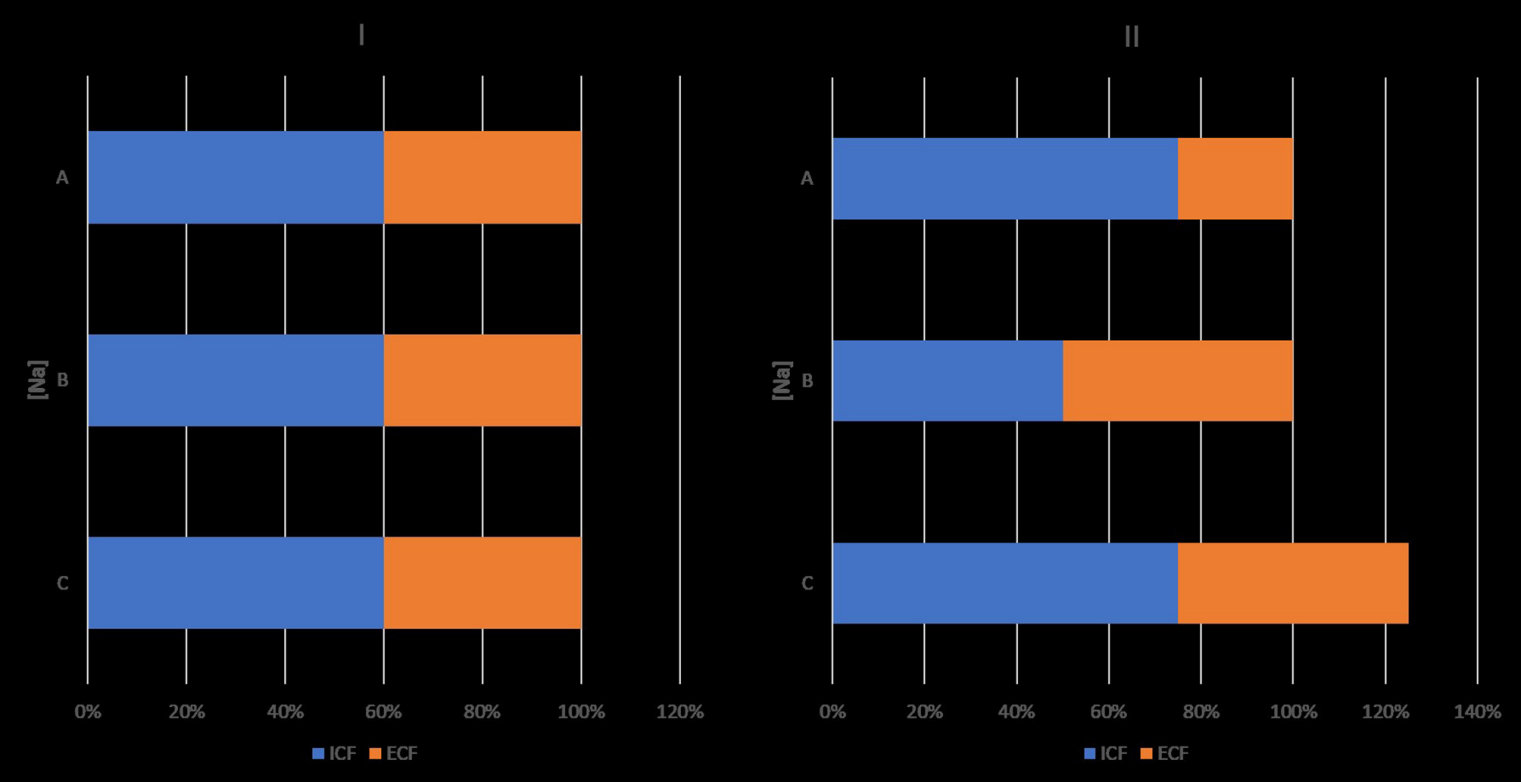
Effects of isolated decreases in total body sodium or total body potassium, and isolated increase in total body water on sodium concentration in serum water and on intracellular and extracellular volumes. Ordinates. sodium concentrations ([Na]). Abscissae: volumes. I: baselines state. II: state after an isolated change. ICV, intracellular volume; ECV, extracellular volume; ICV + ECV = TBW. Product of ordinate and abscissa: amount of monovalent cation in intracellular or extracellular compartment. II, A: decrease in TBNa, which results in hyponatremia, increase in ICV and decrease in ECV. B: decrease in TBK resulting in hyponatremia, decrease in ICV and increase in ECV. C: gain in TBW, which results in hyponatremia and increase in both ICV and ECV, so that their ratio remains the same as in the normonatremic state. Changes in [Na], ICV and ECV are dictated by the Edelman formula, the osmotic principle, and the principle of body water distribution. Hypernatremia secondary to isolated sodium gain is associated with ICV contraction and ECV expansion. Hypernatremia secondary to isolated water loss is associated with proportional ICV and ECV contraction, so that the ICV:ECV ratio is the same as in the normonatremic state. Osmolyte changes for preservation of the normonatremic ICV were not considered in this figure. In clinical practice, isolated changes in one of the components of the Edelman formula are infrequently encountered.

The findings of the Edelman study were consistent with two principles expressing the osmolality of the major fluid compartments in the body and the distribution of TBW between these compartments (8). Peter's osmotic principle, which states that osmolality is equal in the intracellular and extracellular compartments in the steady state (9), was confirmed experimentally more than a decade after its expression (10, 11). The discovery of aquaporins provided the key molecular mechanism of this principle (12, 13). The principle of body water distribution states that TBW is distributed between the intracellular and extracellular compartments in proportion to the amount of solute in each compartment (14). Figure 1, based on formula 1, illustrates the effects of isolated decrease in TBNa or TBK or increase in TBW on intracellular and extracellular volumes and [Na]_SW_.

#### Strengths and Limitations of the Edelman Formula

The use in the statistical analyses of [Na]_SW_ instead of [Na]_S_ constitutes a strength of the Edelman study. Serum has a water phase and a solid phase. Only the water phase contains sodium. Normally, water phase represents 93% of the serum volume and, therefore [Na]_S_ is 93% lower than [Na]_SW._ For example, when [Na]_SW_ is 150 mmol/L, [Na]_S_ will be 150 × 0.93 = 139.5 mmol/L. Measuring sodium concentration by flame photometry or indirect potentiometry results in falsely low [Na]_S_ values, or pseudohyponatremia, in serum samples with higher than normal solid content secondary to hyperlipidemia or hyperproteinemia (15), and in pseudohypernatremia or pseudonormonatremia in serum samples with low solid content secondary to hypoproteinemia (16).

The Edelman study has a few limitations. [Na]_SW_ was not corrected for the degree of glycemia (17). In addition, formula 1, which describes a steady state, provides no information about the time to equilibrium during dynamic periods and does not include factors other than TBNa, TBK, or TBW measured in the steady state that could affect rapidly changing [Na]_SW_ during development or treatment of dysnatremias.

#### The Rose Formula

Rose proposed the following simplified expression of the Edelman formula (18):


(2) $$\begin{array}{l} {\left[Na\right]}_{S}\;=\;\frac{TBNa+TBK}{TBW} \end{array}$$

In the same report, Rose stressed the importance of the relation between urinary losses of water, sodium, and potassium in determining the direction and magnitude of [Na]_S_ change secondary to such losses.

Whether the Rose formula distorts the Edelman formula is a key question. Edelman proposed that formula 1 probably subsumes sodium transfers between osmotically inactive sodium stores in bones and cartilage and osmotically active sodium in body fluids (6), while the Rose formula and all formulas derived from it assume that all the body sodium and body potassium stores are osmotically active. Also, the Rose formula expresses [Na]_S_ while the Edelman formula expresses [Na]_SW_. The clinical laboratories report [Na]_S_.

The Edelman formula would be statistically different from the Rose formula if the 95% confidence interval (CI) for its regression coefficient did not include one or the 95% CI for its y-intercept did not include zero. The 95% CI of the regression coefficient indeed includes one (19) and the 95% CI for the y-intercept is between −5.2 and −46.0 (7, 19). Ring proposed that: (a) accounting for errors in the measurements of TBW, TBNa and TBK could narrow the differences between the Edelman and Rose formulas in both the regression coefficient and the y-intercept; (b) the probability the relation expressed in the Edelman formula is non-linear exists; and (c) the Rose formula may represent our current state of knowledge (19). The Rose formula indicates that the only way to change [Na]_S_ is by changing the fraction (TBNa + TBK)/TBW, while changes in the y-intercept or the regression coefficient of the Edelman formula could also lead to changes in [Na]_SW_ even when the fraction (TBNa + TBK)/TBW does not change.

[Na]_S_ constitutes the key clinical index of body fluid tonicity, or effective osmolarity, which is the property of a fluid to increase, not change, or decrease the volume of cells suspended in it though osmotic fluid shifts (20). The presence of dysnatremia signifies disturbance of tonicity (21–24). Another interpretation of the Edelman and Rose formulas states that tonicity of body fluids equals the fraction of the sum of the main extracellular solute (sodium salts) plus the main intracellular solute (potassium salts) over body water (8, 18, 25). The concepts codified by Edelman have guided the clinical approach to dysnatremias for decades.

### Studies Based on Edelman's Concepts

#### Experimental Studies

Experimental studies verified that changes in the fraction (TBNa + TBK)/TBW determine changes in [Na]_S_ as predicted by Equations (1) and (2). Leaf and co-investigators infused water with dextrose or dextrose and fructose in dogs who either had received vasopressin or had bilateral ureteral ligations or bilateral nephrectomies. In the anuric group, baseline TBW had been measured by deuterium oxide (D_2_O) dilution. In this study of acute hyponatremia, the level of water gain determined the decrease in [Na]_S_ (26). Verbalis showed that combined water retention and sodium losses in the urine determined the degree of hyponatremia in rats injected with a vasopressin analog (27). In the study of Obergaard-Steensen and co-investigators, the changes in TBW, TBNa, and TBK determined the degree of hyponatremia in pigs receiving infusions of vasopressin and 2.5% glucose solution (28).

Infusion of hypertonic saline in anuric dogs verified that TBNa and TBW changes determine the degree of hypernatremia (29–32). Apparent sodium volume of distribution was equal to TBW in these experiments, in agreement with the Edelman report. The formulas comparing the change in [Na]_S_ and the changes in TBNa and TBW in these studies represented modifications of the Rose formula (29, 33).

#### Clinical Reports

The key concept derived from the Edelman report states that a change in [Na]_S_ is mathematically the result of a change, in the same direction, of the fraction (TBNa + TBK)/TBW. Clinical reports addressing various aspects of hyponatremia, including specific categories (34–49), general reviews (50–57), and treatment (58–61), focus their analyses on combinations of losses or gains of TBNa, TBK, and TBW that decrease the fraction (TBNa + TBK)/TBW. Similarly, reports analyzing the pathogenesis and treatment of hypernatremia focus on combinations of losses or gains that increase the fraction (TBNa + TBK)/TBW (62–72).

Renal losses of water, sodium, and potassium may cause dysnatremias. Solute and water excretion in urine were originally expressed by the formulas of osmolal clearance and solute-free water clearance. Urine and serum osmolalities were used in the calculation of these clearances. The formulas of electrolyte (sodium plus potassium) clearance (73–82) and electrolyte-free water clearance (73–81) or water excretion (82) express the Edelman concept during development of dysnatremias secondary to urinary losses. These two formulas are shown below:

Electrolyte renal clearance (C_Na+K_):


(3) $$\begin{array}{l} C_{Na+K}\;=\;V_{U}\times \frac{{\left[Na\right]}_{U}+{\left[K\right]}_{U}}{{\left[Na\right]}_{S}} \end{array}$$

Electrolyte-free renal water clearance (C_H2O(Na+KFree)_):


(4) $$\begin{array}{l} C_{H2O(Na+K\;Free)}\;=\;V_{U}\times (1-\;\frac{{\left[Na\right]}_{U}+{\left[K\right]}_{U}}{{\left[Na\right]}_{S}}), \end{array}$$

where V_U_ = urine flow rate, usually expressed in L or mL per 24 h; [Na]_U_ = urine sodium concentration; and [K]_U_ = urine potassium concentration.

Osmotic diuresis caused by solutes other than sodium salts, e.g., urea, illustrates the clinical application of formulas 3 and 4. In osmotic diuresis, osmolality level is higher in urine than in plasma but the sum of sodium plus potassium concentrations in the urine is lower than [Na]_S_ resulting in hypernatremia (73). The formulas of electrolyte clearance and electrolyte-free water clearance explain accurately the development of hypernatremia in osmotic diuresis, while the formulas of osmolal clearance and solute-free water clearance or excretion fail to do so (73–81).

#### Application of the Edelman Concept in the Treatment of Dysnatremias

Treatment of dysnatremias with clinical manifestations includes infusion of non-isonatric saline or water. Computation of the infusion volumes represents a key application of the Edelman concept. A method for treatment of hyponatremia proposes initial infusion of the same volume of saline to all patients. Several formulas based on the Rose formula and calculating indirectly or directly the volume of the infused saline based on estimates of TBNa, TBK, and TBW have also been proposed (71, 83–86).

The original Adrogué-Madias formula calculates the change in [Na]_S_ after infusion of 1 L of non-isotonic saline using the pre-infusion TBW (TBW_1_) and [Na]_S_ ([Na]_S1_) and the concentration of sodium in the infusate ([Na]_Inf_) (83). This formula was subsequently modified for combined infusion of saline and potassium salts by the addition of potassium concentration in the infusate ([K]_Inf_), as follows (55):


(5) $$\begin{array}{l} {\left[Na\right]}_{S2}-[Na{]}_{S1}\;=\;\frac{{\left[Na\right]}_{Inf}+[K{]}_{Inf}-[Na{]}_{S1}}{TBW_{1}+1} \end{array}$$

where [Na]_S2_ = final serum sodium concentration.

Formula 5 represents an approach to dysnatremias in a closed system according to the Edelman concept, because it incorporates all the factors that will affect the change in [Na]_S_ during infusion of non-isonatric monovalent cation solution, including the volume of the infusate, the initial TBW, and the difference between the concentration of monovalent cations in the infusate and [Na]_S1_. Formulas created subsequently and addressing an open system use the factors included in the Adrogué-Madias formula plus estimates of losses of water, sodium and potassium during treatment of dysnatremias. The importance of infused volume, losses during treatment, and initial TBW to the change in [Na]_S_ will be analyzed below.

The volume of infused non-isonatric saline has relatively small effects on the change in [Na]_S_, but not accounting for this volume can lead to errors (30). The effect of infused volume on the change in [Na]_S_ is proportional to the ratio of infused volume over TBW_1_. For example, infusion of 1 L of 3% saline (sodium concentration of 513 mmol/L) in a patient with TBW equal to 40 L and initial [Na]_S_ 100 mmol/L will produce a final [Na]_S_ equal to 110.1 mmol/L by the Adrogué-Madias formula and 110.3 mmol/L if the infused volume is not included in the calculations. If the same patient had developed hypovolemic hyponatremia with a volume loss of 3.33 L and a [Na]_S_ of 100 mmol/L and received the same load of sodium (513 mmol) in 3.33 L of isotonic saline (154 mmol/L), the final [Na]_S_ will be 110.3 mmol/L using of the Adrogué-Madias formula and 111.3 mmol/L if the infused saline volume is not included in the calculation.

#### Effects of Losses of Sodium, Potassium, and Water During Treatment of Dysnatremias

Patients receiving infusions of non-isonatric solutions exhibit external losses or gains of water, sodium, and potassium, which affect the change in [Na]_S_ (86). Adrogué and Madias proposed a formula calculating the change in [Na]_S_ after the loss of 1 L of body fluids containing sodium and potassium (55). Two formulas calculate the change in [Na]_S_ produced by the infusion of non-isonatric solutions by adding urinary losses of water, sodium and potassium to the determinants of change in [Na]_S_ included in the Adrogué-Madias formula (84, 85).

Studies pertaining to the treatment of dysnatremias provided evidence supporting the role of urinary losses of water and monovalent cations on the change in [Na]_S._ Two studies compared [Na]_S_ values predicted by the Adrogué-Madias formula and observed after non-isonatric saline infusion in dysnatremias (87, 88). In the study of Liamis and coauthors, predicted and observed [Na]_S_ values did not differ statistically 12 h after starting saline infusion for hyponatremia due to hypovolemia, the syndrome of inappropriate vasopressin (SIADH), or diuretic use, but observed values were significantly higher than predicted values after 12 h in hyponatremia due to primary polydipsia, after 24 h in hypovolemic hyponatremia, and after 12 h in hypernatremia (87). Mohmad and co-investigators reported an association between overcorrection of [Na]_S_ by hypertonic saline infusion and very low [Na]_S_ at presentation (111.9 ± 1.5 mmol/L); 40% of the patients developing overcorrection had water diuresis (88). Berl proposed that urinary loss of hypotonic fluid was the main cause of overcorrection of [Na]_S_ in Mohmad's study (89). Overestimation of TBW in patients with hypovolemic hyponatremia may also have contributed to the underestimation of the change in [Na]_S_ in this study.

Urinary losses vary greatly during correction of various types of hyponatremia. Treatment of certain categories of hyponatremia, e.g., hypovolemic hyponatremia, primary polydipsia, or “tea and toast” hyponatremia, leads to large water losses in the urine. Desmopressin infusion prevents excessive rises of [Na]_S_ in these hyponatremias (90, 91). In addition, losses of water, sodium and potassium through the gastrointestinal tract, the lungs, and the skin during treatment of hyponatremias are not predictable and may vary substantially (86). Urinary losses of water, sodium, and potassium during treatment of severe dysnatremias should be measured and included in the calculations of the volume of infused fluids (86). However, frequent measurements of [Na]_S_ represent a key measure during treatment of dysnatremias (86, 92) regardless of the method of treatment.

#### Importance of the Value of Baseline TBW in the Treatment of Dysnatremias

The pre-treatment TBW constitutes a major determinant of the change in [Na]_S_ produced by infusion of non-isonatric saline (53, 70, 86). Treating dysnatremia by infusing the same volume of saline to all patients will result in different changes in [Na]_S_ in patients with different TBW values. Chifu and coinvestigators reported overcorrection of [Na]_S_ in 21% of all hyponatremic patients and 47% of hyponatremic patients with severe symptoms who received the same load of hypertonic saline (93). It is probable that low TBW, along with ongoing losses of fluids during treatment, accounted for this overcorrection.

Estimates of TBW have been applied in the calculation of the volume of infused non-isonatric saline for correcting dysnatremia. Using a fixed fraction of body weight (e.g., 0.6 for men and 0.5 for women) as TBW in these calculations ignores influences of body composition on body water. Lean body mass contains essentially all of body water and body fat contains a negligible part of body water. Table 1 shows two sets of formulas estimating TBW computed by linear regression of TBW measured by isotopic dilution methods on gender, age, height, and weight (94, 95). These formulas have been applied in estimating TBW in various populations, particularly for calculating fractional urea clearance (Kt/V urea) in patients on peritoneal dialysis.

**Table 1:** Anthropometric formulas estimating body water.

Hume and Weyer formulas (94): Women:*TBW* = −35.270121 + 0.344547 × *H* + 0.183809 × *W* Men: *TBW* = −14.012934 + 0.194786 × *H* + 0.296785 × *W*
Watson and coinvestigators formulas (95): Women: *TBW* = −2.097 + 0.1069 × *H* + 0.2466 × *W* Men: *TBW* = 2.447−0.09516 × *A* + 0.1074 × *H* + 0.3362 × *W*

*TBW, total body water; H, height (cm); W, weight (kg); A, age (years)*.

Table 2 shows TBW estimates by various methods and calculations of the increase in [Na]_S_ by the Adrogué-Madias formula in hypothetical patients with normal TBW values and initial [Na]_S_ of 100 mmol/L receiving an infusion of 1 L of 3% saline. TBW was calculated as fixed fractions of body weight and by the anthropometric formulas shown in Table 1. As Table 2 shows infusion of the same volume of saline in subjects with normal TBW, and the same initial [Na]_S_ and weight, but with different age and height, i.e., with different body composition, will result in differing [Na]_S_ changes.

**Table 2:** Body water estimates and rise in serum sodium computed by the Adrogué-Madias formula in subjects with initial serum sodium of 100 mmol/L receiving an infusion of 1 L of 3% saline (513 mmol of sodium).

	**TBW_**1**_, L**	**[Na]_**S2**_ – [Na]_**S1**_**
W, TBW_1_ = 0.5xWeight W, TBW_1_ = 0.6xWeight	35.00 42.00	11.47 9.60
Hume formula (94): W, A = 20 y, H = 180 cm W, A = 60 y, H = 150 cm	39.62 29.28	10.42 13.64
Watson formula (95): W, A = 20 y, H = 180 cm W, A = 60 y, H = 150 cm	34.41 31.20	11.66 12.83
M, TBW_1_ = 0.6xWeight M, TBW = 0.5xWeight	42.00 35.00	9.60 11.47
Hume formula (94): M, A = 20 y, H = 200 cm M, A = 60 y, H = 160 cm	45.72 37.93	8.84 10.89
Watson formula (95): M, A = 20 y, H = 200 cm M, A = 60 y, H = 160 cm	45.56 37.46	8.87 10.74

*TBW_1_, initial total body water; [Na]_S2_, final serum sodium concentration, mmol/L; [Na]_S1_, initial serum sodium concentration, mmol/L; W, women; M, men; A, age; y, years; H, height. Weight was the same in all subjects (70 kg)*.

The differences between subjects with normal TBW receiving the same volume of non-isonatric saline are larger than the values shown in Table 2, which represent average values for each subject category. The changes in [Na]_S_ produced by the same volume of infused saline will be even larger during infusion of saline for treatment of dysnatremias with abnormal TBW, e.g., in patients with hypovolemic or hypervolemic hyponatremia. The anthropometric formulas were developed in studies of normal subjects and tend to systematically underestimate TBW in patients with water excess and to overestimate TBW in patients with water deficit (96). If the weight at normal TBW is known in these patients, the anthropometric formulas can estimate TBW at this normal weight and then the difference between the normal weight and the weight at dysnatremia can be added to or subtracted from the normal TBW to estimate TBW at the dysnatremia episode (86). However, the normal weight is not known in many instances. The estimate of TBW entered in formulas computing the volume of non-isonatric saline required for any given change in [Na]_S_ and the estimate of losses of sodium, potassium and water during treatment represent sources of error potentially much larger than not including the volume of infused saline in the calculations.

The deficiencies of the methods of treating hyponatremia discussed so far indicate aspects that can be improved within the principles of treatment dictated by the Edelman concepts. Areas requiring improvement include estimates of TBW and of losses of water and monovalent cations. The next sections discuss challenges to Edelman's concepts.

### Challenges to Edelman Concept

There is evidence indicating that the relationship between a change in the fraction (TBNa + TBK)/TBW and a change in [Na]_S_ is not always linear. Changes in (TBNa + TBK)/TBW may not be reflected proportionally in changes to [Na]_S_ and part of TBW may not be available for participation in osmotic phenomena.

#### Influences on the Numerator of the Edelman Formula

Total body sodium consists of exchangeable osmotically active and osmotically inactive parts plus a non-exchangeable osmotically inactive store. Exchangeable non-osmotically active sodium is stored in polyanionic proteoglycans found in skin, cartilage, bones (23), muscle, and endothelial surface layers. Sodium exchanges between these tissue stores and body fluids represent a challenge to the approach to dysnatremias when applying formulas based on the Rose formula. Experimental studies performed by Titze and coinvestigators established the existence of non-osmotic storage of sodium in the skin of rats under conditions of excessive salt intake or hormonal (mineralocorticoid) challenge (97–99) and mobilization of sodium from this stored compartment when sodium intake is reduced or when there is a growth spurt (100). Glycosaminoglycan was identified as an important proteoglycan storing sodium in rats (101) and in humans (102).

Balance studies in humans under conditions simulating space station living also established osmotic inactivation of sodium under conditions of high sodium intake (103). Sodium retention is thought to be associated with development of hypertension (104, 105). High sodium intake and non-osmotic storage induces tissue remodeling and activates immune cell homeostasis leading to fibrosis, inflammation, changes in the renal microcirculation and chronic kidney disease, and is associated with cardiovascular risks and comorbidities (106). Osmotic inactivation is considered a mechanism for water conservation (107–109).

The experimental studies, which established the non-osmotic sodium storage in tissue compartments, were performed over long periods of time. The following questions pertinent to dysnatremias are raised: (a) Do sodium exchanges develop between osmotically active and inactive stores during relatively rapid changes of the fraction (TBNa + TBK)/TBW? (b) Can we modify the methods of management of dysnatremias by incorporating interactions between the extracellular osmotically active sodium and the osmotically inactive sodium in tissue stores? And (c) Do disease-induced changes in sodium stores cause dysnatremias without changes in the external balances of sodium, potassium, and water? This third question has not been addressed so far.

Observations during development or treatment of hyponatremia have confirmed the existence of sodium exchanges between its osmotically inactive and active compartments during rapid changes of the fraction (TBNa + TBK)/TBW. Topics of studies which suggested rapid transfer of sodium between osmotically inactive tissue stores and compartments with osmotically active sodium include treatment of the syndrome of inappropriate ADH secretion (SIADH) (37), hyponatremia developing during intense exercise after consumption of water or hypotonic solutions (110, 111), and hyponatremia after use of thiazides (112).

Recently, experimental studies investigated osmotic inactivation after infusion of hypertonic sodium salt solutions. Adrogué and coinvestigators compared apparent sodium volume of distribution and TBW by infusion of hypertonic sodium bicarbonate in three groups of dogs: (a) a group with normal TBW and stores of sodium and potassium; (b) a group which through chronic feeding of hydrochloric acid had modest deficits of water, sodium and potassium, plus metabolic acidosis; and (c) a group with pronounced deficits of water, sodium and potassium plus metabolic alkalosis produced by dietary sodium chloride deprivation and diuretics. Consistent with the Edelman concept, TBW and sodium space were not different statistically in the first and third groups at 30, 60, and 90 min, while in the second group sodium space was significantly greater than TBW in all three time intervals, a finding suggesting osmotic inactivation of part of the infused sodium (113). In a follow-up study using a similar design, Adrogué and collaborators found that apparent sodium volume of distribution is higher by approximately 30% than TBW in dogs with low baseline plasma bicarbonate concentration due to either metabolic acidosis or respiratory alkalosis and that the high computed level of apparent sodium volume of distribution is present at 30 min after infusion of hypertonic sodium bicarbonate and remains at the same level up to 90 min post-infusion (114).

Genetic influences may also impact exchanges of sodium between osmotically inactive and active stores. Heparan sulfate is an essential component of proteoglycans found in cell surface and matrix. Mice with loss of heparan sulfate polymerization genes exhibit an abnormal response to infusion of hypertonic saline, apparently because of defective sodium storage (115). Deficient polymerization of heparan sulfate occurs in humans with hereditary multiple exostosis (HME), who have mutations of the genes EXT-1 and EXT-2 (116). Metabolism of glycosaminoglycans is also altered in patients with type 1 diabetes mellitus, who have low levels of these compounds in several organs due to decreased synthesis and increased destruction (117). A study of hypertonic saline infusion after chronic dietary sodium chloride loading revealed differences between patients with type 1 diabetes mellitus or HME and normal individuals. All three groups exhibited as a response to high salt intake an increase in the expression of nuclear factor of activated T-cells 5 (NFAT5), which is a transcription factor of cellular response to hypertonic states. Healthy subjects had an increase of skin heparan sulfate, HME patients demonstrated an increase in skin dermatan sulfate rather than heparan sulfate, while patients with type 1 diabetes mellitus had no change in their skin proteoglycans after chronic dietary sodium loading and exhibited a rise in [Na]_S_ significantly higher than the control or HME groups after infusion of the same load of hypertonic saline (118). One other issue that will need investigation consists of what portion of intracellular potassium that is osmotically inactive (119) is involved in exchanges with the osmotically active part and participates in the determination of [Na]_SW_ during acute changes of the fraction (TBNa + TBK)/TBW.

The conclusion from the studies discussed in this subsection is that sodium transfer between osmotically inactive tissue stores and body fluids containing osmotically active sodium takes place during development or treatment of dysnatremias and should be taken into account. Osmotic inactivation has a role in other processes, as well, including treatment of hypertension and determining cardiovascular risk (120). The next subsection presents attempts to study the impact of osmotic inactivation during treatment of dysnatremias.

#### Theoretical Studies of the Effects of Tissue Storage of Sodium in Dysnatremias

Whether the Edelman formula (formula 1), which was derived with no information about any change in [Na]_SW_ in the study period, incorporates transfers of sodium between osmotically inactive and active stores during development or correction of dysnatremias has not been clarified. Edelman and co-investigators proposed that bone and cartilage sodium stores could account for the y-intercept in Equation 1 (6). In a series of publications, Nguyen, Kurtz, and collaborators explored theoretically the hypothesis that the Edelman formula accounts for osmotic inactivation and can be used as the basis for formulas for correction of dysnatremias (121–131). These investigators analyzed factors that could be responsible for the following features of formula 1: (a) the y-intercept of −25.6; the factors discussed include osmotically inactive sodium and potassium, the concentration of potassium in plasma water, and other solutes osmotically active including glucose (121, 123, 124). (b) The regression coefficient of 1.11; the factors addressed include the osmotic coefficients of sodium salts and Gibbs-Donnan equilibrium (123). And (c) the complex role of potassium in the pathogenesis of hyponatremia (125). Using the same determinants of [Na]_S_ as the formulas derived from the Rose formula plus the coefficients of the Edelman formula, Nguyen and Kurtz produced formulas expressing various aspects of dysnatremias. These formulas calculate the following features: (a) the volume of the infusate needed to produce a desired change in [Na]_S_ while accounting for gains and losses of water, sodium and potassium during treatment (122, 128); (b) the electrolyte-free urinary water clearance (129); (c) various aspects of fluid imbalance affecting [Na]_S_ in hyperglycemia (128); and (d) the volume of infused water required for a desired change in [Na]_S_ in hypervolemic hypernatremia treated with loop diuretics (130). Note that Nguyen-Kurtz formulas assign a value of 0.93 to serum water content.

Nguyen and Kurtz compared their formulas with formulas based on the Rose formula for treatment of dysnatremias with saline infusion (126, 127). In another report, Nguyen and coauthors compared two linear regressions determining [Na]_SW_ as functions of TBNa, TBK and TBW, which they derived by reanalyzing the data in the studies of Edelman (6) and Boling (7), and attributed theoretically the differences between these two regression formulas to dynamic changes in osmotic storage of sodium and potassium, but concluded that changes in [Na]_SW_ can be predicted from the changes in TBNa, TBK, and TBW even when there are changes in the non-osmotic storage of sodium and potassium (131).

#### Clinical Studies Addressing the Effects of Exchanges Between Osmotically Active and Inactive Sodium Stores During Treatment of Dysnatremias

Nguyen-Kurtz formulas and formulas based on the Rose formula were compared in retrospective investigations (132–134) and prospective studies on treatment of dysnatremias (135–137). Lindner and co-investigators compared final [Na]_S_ ([Na]_S2_) after treatment of hypernatremia in patients admitted to the Intensive Care Unit and [Na]_S2_ values predicted by the Adrogué-Madias formula (formula 5), the Barsoum-Levine formula (84), the Nguyen-Kurtz formula (122), and a simple formula produced by themselves estimating [Na]_S2_ as the sum of two fractions, the fraction (TBNa + TBK)/TBW on admission and the fraction (ΔNa + ΔK)/ΔTBW during treatment, where ΔNa, ΔK, and ΔTBW are the net external changes in the body balances of sodium, potassium, and water during the treatment period. [Na]_S2_ computed from all four formulas correlated with the observed [Na]_S2_. The group-level average differences between measured and predicted [Na]_S2_ were similar among the Adrogué-Madias, Barsoum-Levine, and Nguyen-Kurtz formulas, and lesser for the formula produced by the authors. However, the extent of individual differences between predicted and measured [Na]_S2_ was substantial (132).

Lindner and Schwarz (133) found no significant differences between the electrolyte-free water clearance formula (formula 4, or EFWC) and the modified electrolyte-free water clearance formula produced by Nguyen and Kurtz (129) in predicting [Na]_S_ in critically ill patients with hypernatremia, normonatremia, and hyponatremia. Hanna and coauthors found no differences between the formulas of Adrogué-Madias, Barsoum-Levine, Nguyen-Kurtz, and EFWC in predicting [Na]_S2_ in patients with dysnatremias, but large differences between measured and predicted [Na]_S2_ by every formula (134).

Katsiampoura and co-investigators compared prospectively the Adrogué-Madias, Barsoum-Levine, Nguyen-Kurtz, and EFWC formulas, plus a formula produced by the authors which was based on mass conservation accounting for pre-treatment TBNa, and the amounts of sodium infused, ingested and lost in the urine in patients treated for dysnatremias (135). They found no significant differences between the first four formulas and, based on the comparison of [Na]_S2_ measured and predicted by their mass conservation formula, concluded that this formula is appropriate for predicting [Na]_S2_. Olde-Engberink and co-authors (136) compared the Adrogué-Madias (83) and Nguyen-Kurtz (122) formulas in healthy subjects infused with hypertonic saline after an 8-day low-sodium diet. The observed mean increase in [Na]_S_ 5 min after infusion (3.5 ± 0.4 mmol/L) did not differ from the values predicted by either the Adrogué-Madias formula (3.3 ± 0.1 mmol/L) or the Nguyen-Kurtz formula (3.1 ± 0.1 mmol/L). However, 4 h after infusion [Na]_S_ decreased by 1.8 ± 0.5 mmol/L from its 5-min post-infusion peak value and the urine losses of water, sodium and potassium during that period were not sufficient to account for this decrease. The authors concluded that part of the decrease in [Na]_S_ from its peak post-infusion value was due to non-osmotic storage of sodium (136). One question not addressed in this report is whether the study subjects developed thirst from hypernatremia and consumed fluids during the 4 h post-infusion.

Finally, Wouda and coauthors studied changes in [Na]_S_ in healthy volunteers who drank a standardized volume of water after 8 h of fasting (137). The observed [Na]_S2_ was significantly higher at 120 min post-ingestion than [Na]_S2_ values predicted by both the Barsoum-Levine and Nguyen-Kurtz formulas. However, the lowest observed [Na]_S2_ values were noted 30 min post-ingestion, and [Na]_S2_ increased subsequently up to 120 min post-ingestion. The lowest value of blood hematocrit was noticed 60 min after ingestion. The authors interpreted the rise in [Na]_S_ between 30 and 120 min post-ingestion and the discrepancy between the rise in [Na]_S_ and the decrease in hematocrit between 30 and 60 min post-ingestion as evidence of mobilization of non-osmotically active sodium after acute hypotonicity in healthy individuals. In an accompanying editorial, Adrogué and Madias (138) suggested alternative interpretations of the findings in the study of Wouda, including that all the water ingested had not been absorbed 30 min post-ingestion and further water absorption accounted for the decrease in hematocrit between 30 and 60 min post-ingestion. This last statement was based on a study of water absorption after oral intake by Péronnet and coauthors (139). Another important finding of the Wouda study was a significant discrepancy in monovalent cation rates of urinary excretion between observed values and the values predicted by the Barsoum-Levine or the Nguyen-Kurtz formulas (137). Further studies are needed to clarify the mechanism of the changes in [Na]_S_ noticed after water intake in the study of Wouda.

#### Future Studies Addressing the Accuracy of Formulas in Treating Dysnatremias

Verbalis and coauthors proposed a list of research goals in hyponatremia, including effectiveness and safety of different treatments (140). Formulas offer a quantitative approach to treatment of severe dysnatremias consistent with the Edelman concept. Selection of the proper formula could impact treatment accuracy. However, the studies comparing the formulas for treatment of dysnatremias found essentially no differences between the formulas based on the Rose formula and the Nguyen-Kurtz formulas (132–137). Methodological limitations of these studies could have affected their findings and could have hidden differences in their accuracy.

Future studies of formulas predicting the change in [Na]_S_ during treatment of dysnatremias with any method should have the following characteristics: (a) It is virtually impossible to account for all sources of external changes in sodium, potassium and water during treatment of dysnatremias in retrospective studies. The studies should be prospective. (b) The studies should analyze [Na]_SW_ and not [Na]_S_ (141). Various methods of estimating SWC have been proposed (141–143). If SWC is determined, [Na]_SW_ can be calculated from [Na]_S_ (6). (c) In the studies comparing the various formulas, TBW_1_ was estimated as 0.6 × Weight in men and 0.5 × Weight in women (132, 133), 0.6 × Weight in all patients (134, 136, 137), and 0.5 × Weight in all patients (135). As noted previously (Table 2), TBW_1_ as a fraction of body weight varies even in normal subjects. In addition to the two formulas previously mentioned (94, 95), anthropometric formulas estimating TBW in normal subjects of various ethnicities have been published. Recent reports have cast doubts on the use of clinical algorithms including ethnicity (144, 145). This doubt applies to anthropometric formulas estimating TBW. The determinants of TBW/body weight, including lean body mass, body fat, and difference of TBW from the normal TBW, may be different in each patient with dysnatremia. TBW should be measured in each patient in future studies of dysnatremias. Bioimpedance (BIA) currently represents a suitable method for measuring TBW at presentation with dysnatremia and during its treatment because it is simple, not expensive, non-invasive, and can be repeated frequently. BIA has been used extensively to determine dry weight in patients on dialysis (146, 147) and recorded with reasonable accuracy rapid changes in TBW during the course of hemodialysis sessions (148). However, significant discrepancies between TBW measurements by BIA and isotopic dilution methods have been reported (149). Nguyen and coauthors proposed that serial measurements of exchangeable TBNa, TBK, and TBW by isotopic methods may answer the key question whether [Na]_S_ changes can be predicted in view of the exchanges between osmotic active and inactive sodium stores (150). Further research is needed for developing suitable methods measuring TBW repeatedly in dysnatremias. And (d) during treatment [Na]_SW_ should be monitored and all intakes of water, sodium, and potassium and losses that can be measured should be measured. Improvement of treatment outcomes should be expected by applying point of care for these measurements and incorporating the findings of the measurements in the treatment plan.

#### Influences on the Denominator of the Edelman Formula

Availability of all of body water for osmotic equilibration between body fluid compartments is another issue requiring study. *In-vitro* studies suggest that the unique polar tetrahedral structure of water results in hydrogen binding and hydration shells around intracellular and extracellular proteins and, possibly, even more extended order states such as the crystalline-like exclusion zones at the interface of water and hydrophilic bio-polymers. Water combined with denatured bovine collagen and heat forms gelatin, a semi-solid with five times as much water by weight as the hydrolyzed collagen. This effect is greatly exaggerated in gelatin compared to intact triple helix collagen but even if the phenomenon is highly mitigated in the body, collagen's ubiquitous nature, constituting 20–30% of total body protein (151), suggests the potential for a significant fraction of TBW to be involved in gel formation. To understand the impact of gel formation on the Edelman concepts, both the fraction of TBW involved and the ionic solubility characteristics of bound or structured water must be elucidated.

Hydrogen bonding of water to proteins is well-described but is limited in scope to a few layers of water molecules that form hydration shells or that become trapped when proteins fold to form complex quaternary structures. A water molecule tightly quartered and hydrogen-bound in a protein “pocket” is not likely to take part in a hydration shell around a sodium ion as occurs in bulk or free water. While virtually all proteins are involved in hydrogen bonding with water, experimental evidence suggests that there are other interfacial effects of biopolymers on the physical characteristics of surrounding water in biological tissue. A proposed templating phenomenon has the potential to induce structured or crystalline-like water much more extensive in scope than hydrogen bonded water, potentially extending millions of layers from the interface into adjacent water. This crystalline or ice-like form of water is variously described as the un-mixed layer or exclusion zone (EZ) (152, 153). Hydrophilic polymers with regularly spaced and repeating charged constituents are proposed to act as a template for the polar and tetrahedral water molecules to stack and organize as hexagonal sheets with hydrogens interposed between oxygens (153). The result is higher density, charge separation, reduced pH, and most importantly for our purposes, exclusion of small solutes down to the size of sodium and chloride. Zhang and colleagues reported reductions of sodium concentration by 60% in the supernatant of dilute 1 mM saline solution exposed to Nafion^TM^, a synthetic hydrophilic polymer (154). Biological proteins have hydrophilic and repeating domains that are proposed to function in this manner when interfaced with water. It should be noted that the explanation for the physical findings related to interfacial water chemistry is highly debated. Other possible explanations for the phenomenology described by Pollack (155) and others include ion exchange and diffusiophoresis resulting from ion streams exuded by the synthetic hydrophilic polymers and Van Der Waals forces (156, 157).

Experimental studies of biological water are limited to indirect measurements using infrared and NMR spectroscopy but some observations regarding the cytoplasm are relevant. Intracellular water is part of a viscous and cohesive soup of macromolecules that remains largely intact even when the cell membrane is disrupted. Proteins make up 20–30% of the cytoplasm and macromolecular concentrations are on the order of 200 mg/dL (156). The average distance between macromolecules in the cytoplasm is around 1 nm, corresponding to just three to four molecular layers of water (157). Shi and colleagues (158) used stimulated Raman excited fluorescence microscopy to spatially resolve the distribution of water states inside single mammalian cells. They estimated that bound water makes up 64% of the cytoplasm and 35% of the nucleus in live HeLa cells (a human cancer-derived mammalian cell line). Using NMR spectroscopy, Persson and Halle (159) observed longitudinal water—^2^H spin relaxation times in E. coli cells cultured in D_2_O. By their measure, 10–25% of water molecules in cells have slower re-orientational dynamics, by around an order of magnitude, than those in the bulk. The limited nature of the immobilized water in the setting of close-range protein interactions suggests that hydrogen bonding is at play rather than the extensive, long-distance effects postulated to occur in EZ water formation. Evidence for the more extended effect of EZ water in biological tissue comes from Green and Otori (152), who examined the interface between isolated murine cornea and water with microscopic (0.25 microns) polystyrene latex spheres. In the unstirred state, the spheres were excluded from the vicinity of the cornea for up to 350 micrometers. With stirring the region shrank to 65 microns. Pollack and colleagues later observed similar effects using synthetic polymers and in some biologic materials such as cellulose (160) and observed the other unique physical characteristics, but *in-vivo* data are lacking.

Clearly bulk or amorphous fluid water dominates in biological systems as evidenced by intracellular cytoplasmic streaming and circulation in the lymphatic and vascular systems. That said, hydrogen bonding between proteins and water is likely to limit some fraction of total body water from taking part in ionic solvation. EZ water has the potential for much more extensive effects than hydrogen bonding, but its presence is controversial and *in-vivo* evidence of its presence is lacking. As is the case in much of biology, more research is needed in this under studied area. The volume of water bound to hydrophilic surfaces in body tissues and the changes in hydrophilic binding produced by changes in [Na]_SW_ are currently not known. The quantitative contribution of hydrophilic binding to the changes in [Na]_SW_ during development and treatment of dysnatremias should be subject of investigation.

#### Genetic Influences on Dysnatremias

**Figure 2:**
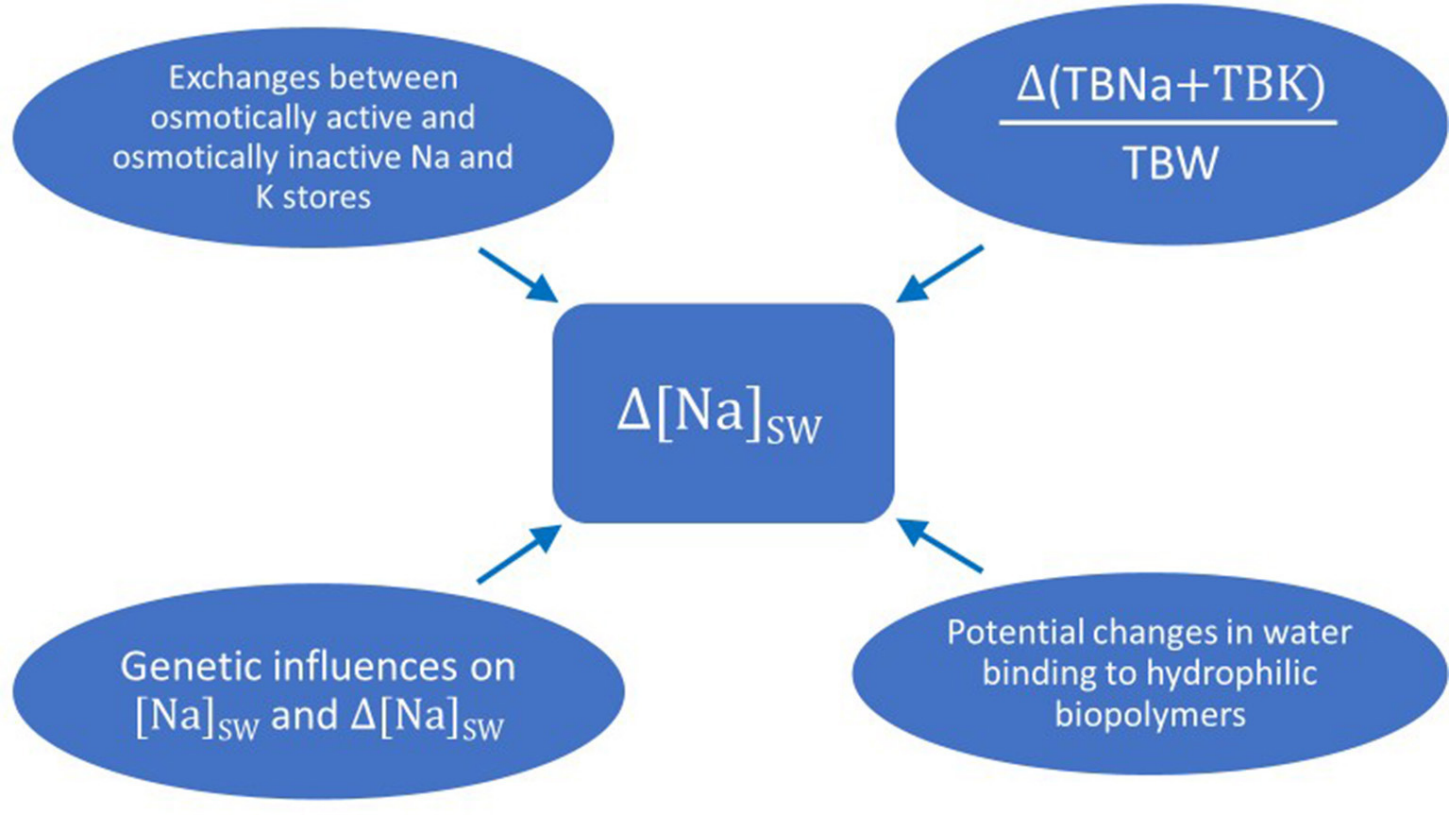
Potential influences on the magnitude of the change in sodium concentration in serum water when there is a change in the fraction (total body sodium plus total body potassium) over total body water. Δ(TBNa + TBK)/TBW: change in the fraction (total body sodium plus total body potassium) over total body water; Δ[Na]_SW_: change in sodium concentration in serum water.

One last issue regarding the pathogenesis and potentially the treatment of dysnatremias is the role of genetic influences on their development (161). The range of normal [Na]_S_ in populations is wide, while individuals have narrow ranges of normal [Na]_S_ (162). Tian and coinvestigators found an association of hyponatremia with a loss-of-function polymorphism of the channel Transient Receptor Potential Vanilloid 4 (TRPV4), which is found in the hypothalamus of mammals and is activated by hypotonic stress (163). Genetic associations with the level of [Na]_S_ in population were subsequently found in two studies (164, 165). Further studies are required to establish the hereditary pathways in regulating [Na]_S_ and their role in the development of dysnatremias. Figure 2 shows the influences that can potentially affect the magnitude of the change in [Na]_SW_ discussed in the previous sections.

## Conclusions

The main concept derived from the Edelman study states that both the pathogenesis and treatment of dysnatremias are consequences of various combinations of external changes in sodium, potassium, and water. This concept has been supported by numerous studies addressing various aspects of dysnatremias. Osmotic inactivation or mobilization of sodium from tissue stores occurs during both development and treatment of dysnatremias and should be accounted for when treating dysnatremias. Calculation of the volume of non-isotonic solutions needed for treating severe dysnatremias requires future well-planned studies. Several basic science questions about the physiologic regulation and the mechanisms of abnormalities of [Na]_S_ require also further studying. Regardless of the method of treatment, monitoring of [Na]_S_ both during and after treatment remains critical. Monitoring of urinary volume and monovalent cation excretion is also needed.

## Author Contributions

MR, AT, and TI: conceptualization. MR, CA, MU, and AT: literature review. MR and AT: methodology and writing—original draft preparation. RS and AT: figure construction. RS, DR, CA, MU, SL, TI, and NL: writing—review and editing. All authors contributed to this article and approved the submitted version.

## Conflict of Interest

The authors declare that the research was conducted in the absence of any commercial or financial relationships that could be construed as a potential conflict of interest.

## Publisher's Note

All claims expressed in this article are solely those of the authors and do not necessarily represent those of their affiliated organizations, or those of the publisher, the editors and the reviewers. Any product that may be evaluated in this article, or claim that may be made by its manufacturer, is not guaranteed or endorsed by the publisher.
